# Sporadic isolation of *Tetratrichomonas* species from the cattle urogenital tract

**DOI:** 10.1017/S003118202100086X

**Published:** 2021-09

**Authors:** Nicholas P. Bailey, Elena Velo-Rego, Robert P. Hirt

**Affiliations:** 1 Newcastle University Bioscience Institute, Cookson Building, Medical School, Newcastle University, Framlington Place, Newcastle-Upon-TyneNE2 4HH, UK; 2 Animal and Plant Health Agency, Staplake Mount, StarcrossEX6 8PE, UK

**Keywords:** Cattle, *Tetratrichomonas*, trichomonad, *Tritrichomonas*, urogenital

## Abstract

*Tritrichomonas foetus* is a venereal trichomonad parasite which causes reproductive issues in cattle. No other trichomonads are known to be urogenital pathogens in cattle, but there are several reports of *Tetratrichomonas* and *Pentatrichomonas* isolates of unclear origin from the cattle urogenital tract (UGT) in the Americas. This study reports the first case of a non-*T. foetus* cattle urogenital trichomonad isolate in Europe. Molecular analysis of the internal transcribed spacer (ITS) 1-5.8S ribosomal RNA-ITS 2 and 18S ribosomal RNA loci suggest that the isolate is a *Tetratrichomonas* species from a lineage containing other previously described bull preputial isolates. We identified close sequence similarity between published urogenital and gastrointestinal *Tetratrichomonas* spp., and this is reviewed alongside further evidence regarding the gastrointestinal origin of non-*T. foetus* isolates. Routine screening for *T. foetus* is based on culture and identification by microscopy, and so considering other trichomonad parasites of the bovine UGT is important to avoid misdiagnosis.

## Graphical abstract

**Figure:**
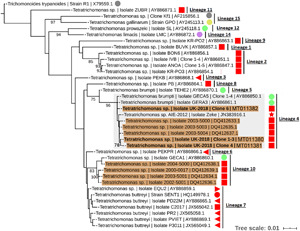

## Introduction

*Tritrichomonas foetus* is an important bovine venereal parasite which causes reproductive issues including spontaneous abortion, pyometra and infertility (Dąbrowska *et al*., 2019), imposing a significant economic burden on the beef and dairy industry (Yule *et al*., 1989). *Tritrichomonas foetus* is also a prevalent cause of diarrhoea in cats (Asisi *et al*., 2008), and is considered to be synonymous with the porcine gut-associated *Tritrichomonas suis* (Slapeta *et al*., 2012). *Tritrichomonas foetus* is a protozoan parasite of the class Tritrichomonadea, which together with Trichomonadea belong to the informal taxonomic group the trichomonads, within the phylum parabasalia (Cepicka *et al*., 2010; Noda *et al*., 2012). *Tetratrichomonas* is a diverse genus within Trichomonadea, which most commonly form symbiotic relationships within the gastrointestinal tract (GIT) of animals including invertebrates, fish, mammals, reptiles, amphibians and birds (Cepicka *et al*., 2006). The genus was originally defined morphologically with characteristics including an ovoid cell body and four anterior flagella of unequal length (Honigberg, 1963). There have been conflicting reports regarding the monophyly of *Tetratrichomonas*, with some molecular phylogenies placing *Trichomonas*, *Pentatrichomonas* and *Trichomonoides* as ingroups (Cepicka *et al*., 2006).

Trichomonads appear to undergo frequent cross-species transmission and transfer to alternative body sites; *Pentatrichomonas hominis* has been isolated from a wide range of mammals including humans, primates, cats, dogs and bovids (Li *et al*., 2016; Bastos *et al*., 2018; Li *et al*., 2020), and it is thought that the human pathogens *Trichomonas tenax* and *Trichomonas vaginalis* arose from cross-species transmission of independent lineages of avian oral parasites to the human mouth and urogenital tract (UGT), respectively (Maritz *et al*., 2014; Peters *et al*., 2020). Some reports have suggested a degree of fidelity between *Tetratrichomonas* and host lineages. For example, Cepicka *et al*. (2006) identified 16 *Tetratrichomonas* lineages of which the majority were fairly limited in the taxonomic range of host species. However, *Tetratrichomonas* spp. are often not limited to a single host, for example, *Tetratrichomonas gallinarum* has been identified as a pathological agent in a wide range of bird species (Cepicka *et al*., 2005). Transmission across wider taxonomic ranges are also not unknown, and reports of *Tetratrichomonas* spp. in the lungs of immunocompetent patients with pulmonary disease (Lin *et al*., 2019) illustrates the potential importance of *Tetratrichomonas* zoonosis for human health.

There have been several reports of non-*Tt*. *foetus* trichomonads isolated from the bull preputial cavity. Morphological and phylogenetic methods have identified these as *P hominis*, a *Pseudotrichomonas* sp. and *Tetratrichomonas* spp., some of which may correspond to the previously described species *Tetratrichomonas buttreyi* (Dufernez *et al*., 2007). The most common method for *Tt. foetus* diagnosis is *in vitro* culture of preputial washings, and subsequent examination of cultures by light microscopy (Parker *et al*., 2003), and so false positives resulting from non-*Tt. foetus* trichomonads may be an issue (Dufernez *et al*., 2007).

This study provides an additional report of a *Tetratrichomonas* sp. isolated from bull preputial washings in the UK, and reviews previous reports on trichomonad species in the bovine UGT.

## Materials and methods

### Source of isolates

Samples were collected from bulls in the UK during routine screening for parasites, in accordance with the principles defined in the European Convention for the Protection of Vertebrate Animals used for Experimental and Other Scientific Purposes. To collect samples, the preputial cavity was washed with 30 mL pre-warmed phosphate buffered saline (PBS) (pH 7.2). Washings were pelleted by centrifugation at 300 × ***g*** for 10 min, suspended in 5 mL PBS and examined for motile protozoa. Parasites were cultured by inoculating 1 mL of the PBS suspension into 10 mL Clausens medium [20 g L^−1^ Neopeptone, 10 g L^−1^ Lab Lemco (Oxoid), 5 g L^−1^ neutralized liver digest (Oxoid) and 20 g L^−1^ glucose, pH 7.4], supplemented with 200 units mL^−1^ penicillin, 200 *μ*g mL^−1^ streptomycin and 1000 units mL^−1^ polymixin B, overlaid on a solid medium slope prepared by heating 7 mL horse serum at 75°C for 2 h. Cultures were incubated at 37°C for 7 days, and were examined microscopically on days 4 and 7 for motile protozoa. Positive cultures were passaged in fresh media, and 10 mL culture was harvested by centrifugation at 300 × ***g*** for 10 min, fixed in 15 mL 100% ethanol and stored at −20°C. No cryopreserved stock of the isolate was generated.

### Molecular sequence typing

Genomic DNA was extracted from ethanol-fixed parasite isolates using the DNeasy ultraclean microbial kit (Qiagen) according to the manufacturer's instructions. Loci for molecular sequence typing were amplified by polymerase chain reaction (PCR) using generic Taq polymerase (NEB). A region containing the 5.8S ribosomal RNA (rRNA) and flanking internal transcribed spacers (ITS) 1 and 2 was amplified using the trichomonad-specific TFR1 and TFR2 primers, and the 18S rRNA gene was amplified using the generic eukaryotic primers Euk 1700 and Euk 1900. Resulting products were cloned into pCR4TOPO using the TOPO TA cloning kit (ThermoFisher Scientific) according to the manufacturer's instructions. Sequences were generated for the inserts from five independent clones by Sanger sequencing (Eurofins) on both strands using the T7 and T3 promoter primers, and additional internal sequencing primers were designed to cover the full length of the 18S rRNA gene for both strands. All primer sequences are listed in Supplementary Table S1.

### Phylogenetic analysis

A reference collection of ITS1-5.8S rRNA-ITS2 and 18S rRNA trichomonad sequences were selected from the NCBI RefSeq database (O'Leary *et al*., 2016) by BLASTn search (Altschul *et al*., 1990) using the new sequences as a query, and from the literature. Sequences were aligned using Tcoffee (Notredame *et al*., 2000), gaps and poorly aligned regions were trimmed automatically using trimAl (Capella-Gutiérrez *et al*., 2009) and resulting alignments were visually inspected for accuracy using Seaview (Gouy *et al*., 2010). Iqtree (Nguyen *et al*., 2015; Kalyaanamoorthy *et al*., 2017) was used to construct maximum likelihood (ML) phylogenies using automatic model selection, and reliability was assessed with 1000 bootstrap replicates. Figures were generated using iTol (Letunic and Bork, 2019). Alignments used to generate phylogenies are available in the Supplementary data (S1, S2 and S3).

### Metatranscriptomics data analysis

Sequence data for total RNA metatranscriptomics sequencing of the cattle rumen was downloaded from NCBI's SRA database (Leinonen *et al*., 2011), selecting a subset of 14 out of a total of 48 available samples for analysis (accessions SRX5208721–SRX5208732 and SRX5229555). SortMeRNA (Kopylova *et al*., 2012) was used to align the reads to the default reference rRNA database, and aligned reads were searched for parasite sequences by BLASTn (Altschul *et al*., 1990), collecting only the top hit for each query.

## Results

Between 2014 and 2021, preputial samples from 9637 bulls were screened for trichomonads. For only a single sample, from a 1-year-old bull in South West England, motile, trichomonad-like protozoa were isolated. Morphological examination by light microscopy revealed four anterior flagella. Rearing conditions of the bull are likely to have allowed sexual contact with other bulls and cows.

In order to identify the isolate, sequences were generated for five clones for the 18S rRNA and ITS1-5.8S rRNA-ITS2 loci. For the 18S rRNA locus, three unique clonal sequences were generated which shared 99.5–99.7% identity, suggesting a single-species infection. ML analysis of the 18S rRNA locus placed all clone sequences together within *Tetratrichomonas* with strong bootstrap support (Supplementary Fig. S1).

For the ITS1-5.8S rRNA-ITS2 locus, there were three unique sequences which shared 95.2–99% identity, suggesting some diversity amongst the parasites present. In agreement with the 18S rRNA locus, ML analysis also placed all the isolates together within *Tetratrichomonas* with moderate bootstrap support (Supplementary Fig. S2). The isolates were also placed in a separate lineage from urogenital *Tetratrichomonas* isolates from cattle originating from a previous study (accession AF342742).

**Fig. 1.**
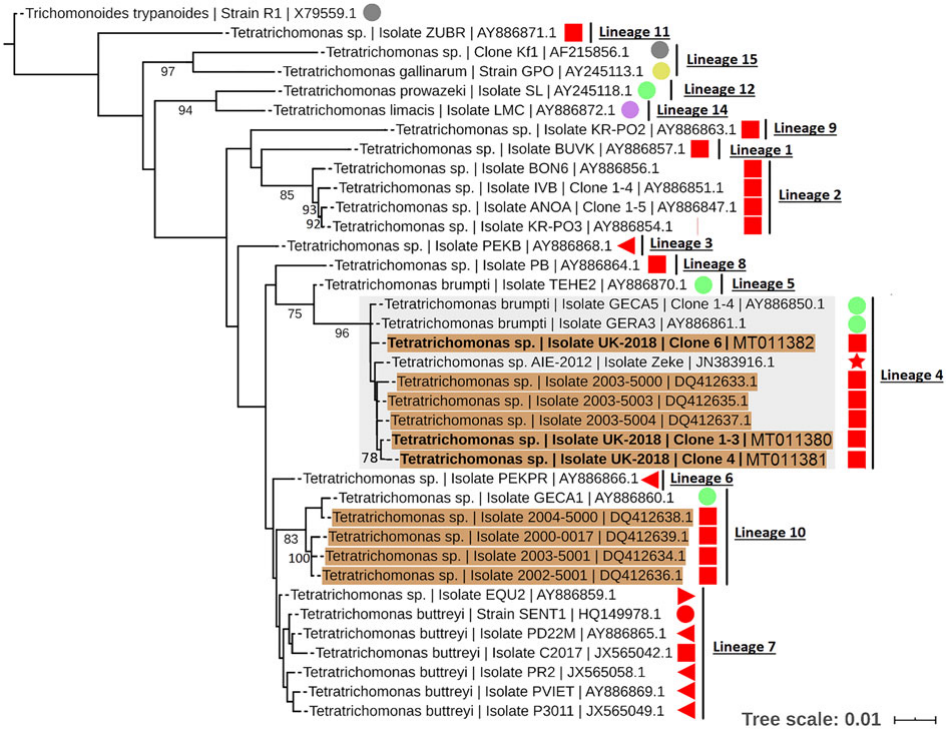
ML phylogeny (GTR model with empirical base frequencies, invariable sites and discrete gamma model) for *Tetratrichomonas* spp. based on the 18S rRNA locus, rooted using *Trichomitus batrachorum* as an outgroup. Bootstrap values (1000 replicates) >70% are shown on branches. New sequences generated in this study are highlighted in bold. *Tetratrichomonas* lineages as defined by Cepicka *et al*. (2006) are annotated, and the lineage of interest is highlighted. Urogenital isolates are highlighted in orange, and host species are annotated with shapes at the tip labels; insect (grey), mollusc (purple), bird (yellow), reptile (green) and mammal (red). Mammals are subdivided into families; bovine (square), porcine (left triangle), equine (right triangle), primate (circle) and anteater (star). Units for tree scale are inferred substitutions per base pair. Genbank (Benson *et al*., 2015) accessions for each sequence are shown at the end of tip labels.

To resolve the *Tetratrichomonas* lineage of the isolates in more detail, ML analysis was performed on the 18S rRNA sequence from a greater taxonomic sampling of *Tetratrichomonas* spp., including several bull urogenital isolates from previous studies (Fig. 1). The sequences for all clones grouped within *Tetratrichomonas* lineage 4 as defined by Cepicka *et al*. (2006), which includes one group of bull urogenital isolates previously reported (accession DQ412633).

In order to investigate the potential gastrointestinal origin of urogenital *Tetratrichomonas* spp. in cattle, we searched published metatranscriptomics data from the cattle rumen for sequences similar to two hypervariable regions (V4 and V8) amongst *Tetratrichomonas* 18S rRNA sequences. For the published urogenital *Tetratrichomonas* isolate 2004–5000 (Dufernez *et al*., 2007; accession DQ412638) we identified sequences identical to the V8 region, and virtually identical (2 base pair mismatch) to the V4 region. BLAST search results were identical in terms of mismatches, gaps and query coverage for 11 out of 14 samples tested. In contrast, we did not identify any sequences of a similarly highly sequence identity to the urogenital *Tetratrichomonas* sequence generated in this study. Representative BLAST results for a single sample are shown in Supplementary Table S2, and an alignment between metatranscriptome sequences and urogenital *Tetratrichomonas* 18S rRNA sequences are shown in Supplementary file S4.

In addition to the isolates identified in this study, there have been numerous reports of trichomonads isolated from the UGT of cattle, summarized in Table 1. A total of 151 trichomonad isolates have been reported. With the exception of isolates obtained from this study, all previous isolates originate from the Americas. Various morphological and molecular techniques have assigned the isolates as *Tetratrichomonas* and *Pentatrichomonas* spp., which appear to have been isolated in roughly equal proportions. In contrast, there has only been a single report of a *Pseudotrichomonas* isolate.

**Table 1.** Summary of reports of non-*Tritrichomonas foetus* trichomonads isolated from the cattle UGT

Reference	Number of isolates	Location	Identification methods^a^
Dufernez *et al*. (2007)	*Tetratrichomonas* 7 *Pentatrichomonas* 4 *Pseudotrichomonas* 1 Total: 12	USA	Morphology, PCR, molecular phylogeny
Kleina *et al*. (2004)	*Tetratrichomonas* 1 *Pentatrichomonas* 0 *Pseudotrichomonas* 0 Total: 1	Brazil	Molecular phylogeny
Walker *et al*. (2003)	*Tetratrichomonas* 14 *Pentatrichomonas* 18 *Pseudotrichomonas* 0 Total: 32	USA	Morphology, sequence comparison
BonDurant *et al*. (1999)	*Tetratrichomonas* 14 *Pentatrichomonas* 0 *Pseudotrichomonas* 0 Total: 14	USA	Morphology, PCR
Campero *et al*. (2003)	*Tetratrichomonas* NA *Pentatrichomonas* NA *Pseudotrichomonas* NA Total: 48	Argentina	Morphology, PCR
Hayes *et al*. (2003)	*Tetratrichomonas* 16 *Pentatrichomonas* 21 *Pseudotrichomonas* 0 Total 37	USA	PCR
Cobo *et al*. (2003)	*Tetratrichomonas* 6 *Pentatrichomonas* 0 *Pseudotrichomonas* 0 Total: 6	Argentina	Morphology, PCR
Parker *et al*. (2003)	NA	Canada	Morphology, PCR
This study	*Tetratrichomonas* 1 *Pentatrichomonas* 0 *Pseudotrichomonas* 0 Total: 1	UK	Morphology, molecular phylogeny
Total	*Tetratrichomonas* 59 *Pentatrichomonas* 43 *Pseudotrichomonas* 1 Total: 151		

^a^PCR and phylogenetic data are based on amplification of the ITS1-5.8S rRNA-ITS2 locus, except for Dufernez *et al*. (2007) and this study, which also examined the 18S rRNA locus.

## Discussion

This study reports the first case of a non-*Tt. foetus* trichomonad isolated from the bovine UGT in Europe. In agreement with previous reports of cattle urogenital trichomonads (Walker *et al*., 2003; Kleina *et al*., 2004; Dufernez *et al*., 2007), the morphological and sequence data indicate that this isolate is a *Tetratrichomonas* sp. The phylogenies presented here are largely in agreement with previous studies regarding the interrelationship between trichomonads and the reported lineages of *Tetratrichomonas* spp. (Cepicka *et al*., 2006; Dufernez *et al*., 2007). Similarly to previous reports (Cepicka *et al*., 2006, 2010), there is discrepancy between phylogenies based on the 18S rRNA and ITS1-5.8S rRNA-ITS2 in terms of the monophyly of the *Tetratrichomonas* genus.

Our results support the placement of the new isolates in *Tetratrichomonas* lineage 4 as defined by Cepicka *et al*. (2006), which corresponds to group B of the bull urogenital *Tetratrichomonas* isolates defined by Dufernez *et al*. (2007). The new isolates were distinct from bull urogenital group A (Dufernez *et al*., 2007), corresponding to *Tetratrichomonas* lineage 10 (Cepicka *et al*., 2006) and also possibly the bull urogenital isolates described by Walker *et al*. (2003), although this is not well resolved. In addition, the ITS1-5.8S rRNA-ITS2 data suggest some genetic diversity amongst the clonal sequences from the new isolate, as the three clones did not cluster together, potentially indicating a non-clonal infection or heterogeneity amongst multi-copy rRNA genes (Torres-Machorro *et al*., 2010). These observations overall suggest a degree of genetic heterogeneity amongst cattle urogenital *Tetratrichomonas* isolates.

*Tetratrichomonas* lineage 4 was described as mostly limited to the tortoise GIT (Cepicka *et al*., 2006). This lineage has also been found in mammals, but excluding the cattle isolates, all others including the elephant isolate SLON (Cepicka *et al*., 2006) and the anteater isolate Zeke (Ibañez-Escribano *et al*., 2013), originate from zoo animals which may suggest an artificial context for transmission. Isolates from *Tetratrichomonas* lineage 10 (Cepicka *et al*., 2006) have also been found in the bull UGT, and based on morphological data it has been suggested that this corresponds to the previously described cattle gastrointestinal species *Te. buttreyi* (Dufernez *et al*., 2007). Intriguingly, *Tetratrichomonas* lineage 10 also appears to be mostly restricted to tortoises (Cepicka *et al*., 2006).

At least two *Tetratrichomonas* lineages, *P. hominis*, and a *Pseudotrichomonas* sp. have been isolated from the cattle UGT (Walker *et al*., 2003; Dufernez *et al*., 2007). These trichomonad isolates alternatively represent either emerging or established urogenital species or sporadic spillover of microbes from an alternative reservoir, most likely the bovine GIT. The possibility of cross contamination during sample collection should also not be discounted for some isolates. The genetic heterogeneity amongst cattle urogenital isolates may support the spillover hypothesis. This is in contrast to parasite species thought to have emerged through cross-species or cross-mucosa transmission, such as *Tt. foetus*, which shows a remarkable degree of genetic homogeneity suggestive of a recent founder event (Kleina *et al*., 2004). Cattle urogenital trichomonad isolation shows a sporadic geographic pattern (Campero *et al*., 2003; Kleina *et al*., 2004; Dufernez *et al*., 2007) which also supports the spillover hypothesis, as the events appear to be unrelated to one another. We identified sequences highly similar to cattle urogenital *Tetratrichomonas* 18S rRNA (Dufernez *et al*., 2007) frequently occurring in the cattle rumen metatranscriptome (Li *et al*., 2019), providing strong evidence that these urogenital isolates are of gastrointestinal origin. The infrequent urogenital detection of non-*Tt. foetus* trichomonads in a backdrop of frequent *Tt. foetus* monitoring (Yao, 2013) suggests that the events are rare, although misidentification of other trichomonads as *Tt. foetus* is also possible.

There are plausible sources for the isolated urogenital trichomonads from the bovine GIT; *Tetratrichomonas* spp. (Zhang *et al*., 2020), such as *Te. buttreyi* (Dufernez *et al*., 2007), are known to inhabit the cattle gut, and *P. hominis* is known to occupy the GIT of a very wide range of animals (Li *et al*., 2016), including cattle (Li *et al*., 2020). The isolate related to the putatively free-living *Pseudotrichomonas keilini* (Dufernez *et al*., 2007) at most represents a very rare event, as only a single isolate has been reported, and so contamination cannot be ruled out. Alternatively, *Monoceromonas ruminantium* is also relatively closely related (Dufernez *et al*., 2007) and has been isolated from the GIT of cattle (Hampl *et al*., 2004), suggesting that the lineage could be host-associated and that *P. keilini* is an exception or mislabelled as free-living. The presence of gastrointestinal-associated bacteria at the same site as urogenital trichomonad isolation provides further evidence for their gut origin (Cobo *et al*., 2003). A route of gastrointestinal to urogenital transmission through sexual mounting behaviour, potentially between young bulls, has been suggested (BonDurant *et al*., 1999).

During experimental inoculation of *Tetratrichomonas* preputial isolates in the cattle UGT, results have ranged from no persistence (Cobo *et al*., 2004) to sporadic re-detection up to 2 weeks after inoculation in mature bulls (Cobo *et al*., 2007*b*) and no persistence beyond 6 h in young heifers (Cobo *et al*., 2004, 2007*a*). *Pentatrichomonas hominis* derived from the cattle gut also failed to persist in the UGT of heifers (Cobo *et al*., 2007*a*). Variation in results is likely due to strain and host differences, however failure of preputial *Tetratrichomonas* to regularly establish colonization during experimental infection (Cobo *et al*., 2004, 2007*a*, 2007*b*) provides strong evidence for the sporadic origin hypothesis. There was no evidence for pathology caused by any of the non-*Tt. foetus* trichomonads (Cobo *et al*., 2004).

Together, the evidence supports the hypothesis that non-*Tt. foetus* trichomonad isolates obtained from the cattle UGT do not represent an emerging urogenital inhabitant but rather a sporadic transmission from another source, most likely the gut. However, transmission of parasites between these mucosal sites may increase the possibility of new pathogens emerging, as has been documented for other trichomonads (Maritz *et al*., 2014). The detection of trichomonads in the cattle UGT, and the mosaic pattern of host and mucosal site specialization amongst the trichomonads highlight their adaptability and thus zoonotic potential.

The most significant concern associated with non-*Tt. foetus* trichomonads in the cattle UGT is most likely misdiagnosis through confusion with *Tt. foetus*, which could cause unnecessary culling of suspected infected animals (Campero *et al*., 2003). The scale of misdiagnosis is unclear; the low detection frequency of trichomonads, particularly in regions which are intensely monitored, such as the UK, suggests a low frequency. However, detection rates were as high as 8.5% in some groups of bulls (Campero *et al*., 2003), suggesting the issue may be more significant in some regions. The most common method of *Tt. foetus* monitoring is culture-based isolation and morphological identification by light microscopy (Parker *et al*., 2003) which may lack specificity because expertise to differentiate trichomonads based on morphology is rare (Dufernez *et al*., 2007). Molecular detection methods offer the advantages of improved speed and specificity (Felleisen, 1997; Hayes *et al*., 2003; Parker *et al*., 2003) and enhanced sensitivity compared with culture-based detection (Cobo *et al*., 2007*b*), without the need for morphological expertise. However, molecular methods are not routinely used due to high cost and impracticality in an agricultural setting. Lower cost molecular methods such as loop-assisted isothermal amplification have been applied to achieve very sensitive and specific *Tt. foetus* detection (Oyhenart *et al*., 2013) and so may offer a more practical solution. As evidence suggests *Tetratrichomonas* sp. are short-lived in the bull UGT (Cobo *et al*., 2004, 2007*b*), and may originate from sexual mounting behaviour (Walker *et al*., 2003), separating bulls before testing may also reduce misdiagnosis. Artificial insemination combined with regular monitoring of semen for parasites has proven a very successful control method (Dąbrowska *et al*., 2019), and should also be considered for more widespread adoption.

## Data Availability

All sequences generated in this study can be obtained from the NCBI Genbank database (Benson *et al*., 2015) under accessions MT011380-MT011382 for the 18S rRNA sequences and MT375127-MT375129 for the ITS1-5.8S rRNA-ITS2 sequences.

## Supplementary material

For supplementary material accompanying this paper visit https://doi.org/10.1017/S003118202100086X. click here to view supplementary material
