# Supplementary material for: Sporadic isolation of Tetratrichomonas species from the cattle urogenital tract
Source: Parasitology. 2021 Jun 3;148(11):1339–44. doi: 10.1017/S003118202100086X (PMC8383267; doi:10.1017/S003118202100086X)
Supplement: Supplementary file 1 [file S003118202100086Xsup001.docx]

**Supplementary material**

**Figure S1.** Unrooted maximum likelihood phylogeny (TVM model with empirical base frequencies, invariable sites and discrete gamma model) for parabasalia based on the 18S rRNA locus. Bootstrap values (1000 replicates) greater than 70% are shown on branches. New sequences generated in this study are highlighted in bold and sequences originating from bovine hosts are marked with an asterisk. Major classes are annotated using guidance from Malik *et al.* (2011) and Cepicka *et al.* (2010), and the lineage of interest is highlighted. Units for tree scale are inferred substitutions per base pair. Genbank (Benson *et al.*, 2015) accessions for each sequence are shown at the end of tip labels.

**Figure S2.** Maximum likelihood phylogeny (TN model with empirical base frequencies and discrete gamma model) for the trichomonads based on the ITS1-5.8S rRNA-ITS2 locus. The root was placed at the branch separating Trichomonadida and Tritrichomonadida according to Malik *et al.* (2011). Bootstrap values (1000 replicates) greater than 70% are shown on branches. New sequences generated in this study are highlighted in bold and sequences originating from bovine hosts are marked with an asterisk. Major classes are annotated according to Cepicka *et al.* (2010), and the lineage of interest is highlighted. Units for tree scale are inferred substitutions per base pair. Genbank (Benson *et al.*, 2015) accessions for each sequence are shown at the end of tip labels.

**Table S1.** Oligonucleotide primers used in this study. Sequences are shown in the 5’-3’ direction.

| Name* | Forward primer sequence | Reverse primer sequence | Reference |
| --- | --- | --- | --- |
| TFR1/2 | TGCTTCAGTTCAGCGGGTCTTCC | CGGTAGGTGAACCTGCCGTTGG | Felleisen (1997) |
| Euk 1700/Euk 1900 | CBGCAGGTTCACCTAC | AYYTGGTTGATYCTGCCA | Gerbod et al. (2001) |
| T7/T3 promoter | TAATACGACTCACTATAGGG | ATTAACCCTCACTAAAGGGA | Commercial |
| 18S rRNA internal | GCGGTAATTCCAGCTCTGC | CCCGAAGCCTGTCAGTCATA | This study |

* name is shown with forward primer of the left and reverse primer on the right

**Table S2.** Representative BLAST alignment results for *Tetratrichomonas* 18S hypervariable regions V4 and V8 (Hadziavdic *et al.*, 2014) against cattle gastrointestinal metatranscriptome data.

| Query | Subject Sample Accession | Subject Sequence ID | Percentage Identity | Query Length | Percentage Query Coverage | Mismatch | Gaps | E value | Bit Score |
| --- | --- | --- | --- | --- | --- | --- | --- | --- | --- |
| Tetratrichomonas sp. \| Isolate UK-2018 \| Clone 1-3 \| MT011380 \| V4 | SRX5208720 | 34787211 | 86.0 | 97 | 100 | 6 | 6 | 1.43E-19 | 100 |
| Tetratrichomonas sp. \| Isolate UK-2018 \| Clone 1-3 \| MT011380 \| V8 |  | 31120191 | 94.4 | 90 |  | 1 | 4 | 3.51E-30 | 135 |
| Tetratrichomonas sp. \| Isolate 2004-5000 \|DQ412638 \| V4 |  | 34669055 | 97.3 | 74 |  | 2 | 0 | 1.62E-27 | 126 |
| Tetratrichomonas sp. \| Isolate 2004-5000 \|DQ412638 \| V8 |  | 34975273 | 100.0 | 74 |  | 0 | 0 | 7.50E-31 | 137 |

**Supplementary data file S1.** Trimmed alignment used to generate phylogeny in Fig. 1

**Supplementary data file S2.** Trimmed alignment used to generate phylogeny in Fig. S1

**Supplementary data file S3.** Trimmed alignment used to generate phylogeny in Fig. S2

**Supplementary data file S4.** Alignment between representative cattle gastrointestinal metatranscriptomics sequences and the V4 and V8 regions of 18S rRNA genes from cattle urogenital *Tetratrichomonas* isolates, alongside a range of 18S rRNA sequences from *Tetratrichomonas* spp. The approximate positions of the V4 and V8 hypervariable regions are indicated at the bottom of the alignment.

**References**

**Hadziavdic K, Lekang K, Lanzen A, Jonassen I, Thompson EM and Troedsson C** (2014) Characterization of the 18S rRNA gene for designing universal eukaryote specific primers. *PLoS One* **9**, e87624.

**Malik SB, Brochu CD, Bilic I, Yuan J, Hess M, Logsdon JM and Carlton JM** (2011) Phylogeny of parasitic parabasalia and free-living relatives inferred from conventional markers vs Rpb1, a single-copy gene. *PLoS One* **6**, 1–14. doi: 10.1371/journal.pone.0020774.
